# A Workup Protocol Combined with Direct Application of Quantitative Nuclear Magnetic Resonance Spectroscopy of Aqueous Samples from Large-Scale Steam Explosion of Biomass

**DOI:** 10.1021/acsomega.0c05642

**Published:** 2021-03-02

**Authors:** Camilla Løhre, Jarl Underhaug, Rune Brusletto, Tanja Barth

**Affiliations:** Department of Chemistry, University of Bergen, Allégt. 41, Bergen 5007, Norway

## Abstract

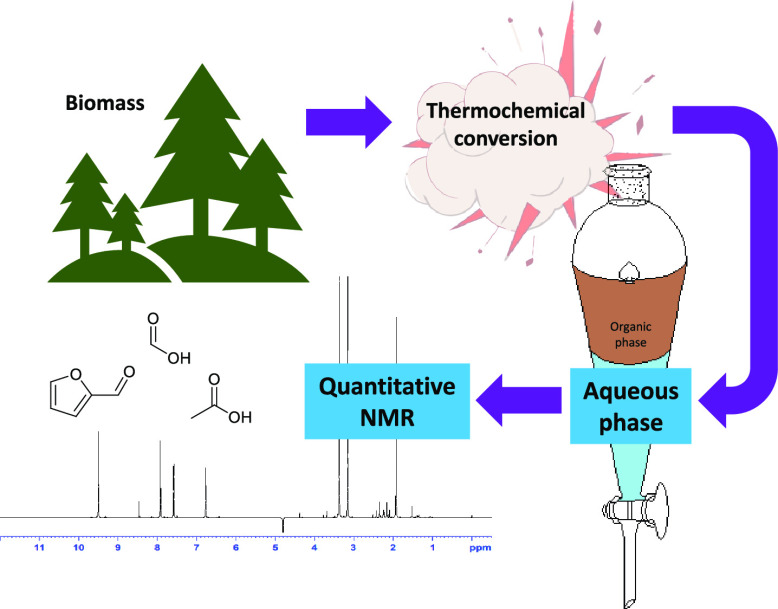

Methods for thermochemical conversion of biomass into renewable energy and materials rapidly increase in range and outreach. A focus on the target product streams for valorization is natural, yet several pretreatment steps and conversion methods also result in an aqueous byproduct, which has been given less attention. This paper aims to fill this knowledge gap in the existing literature on identification and quantification of organic components in such aqueous phases by reporting a fast and direct workup protocol combined with application of quantitative analytical nuclear magnetic resonance (NMR) spectroscopy. Laboratory workup procedures combined with subsequent proton NMR spectroscopy with water signal suppression using presaturation pulses during relaxation delay, *noesygppr1d*, have been established, evaluated, and approved by testing on three different Bruker BioSpin NMR spectrometers; an 850 MHz AVANCE III HD with a 5 mm TCI CryoProbe, a 600 MHz AVANCE NEO with a QCI CryoProbe, and a 500 MHz AVANCE with a 5 mm BBO room-temperature probe additionally confirmed the quantification method to be applicable. The analytical procedure identified furfural, methanol, acetic acid, and formic acid as the dominating compounds in the analyzed aqueous samples, which were process effluents generated by the patented Arbacore pellet production process using steam explosion of wood shavings. A selected range of quantitative results in the aqueous phase from large-scale steam explosion is included in the study. The described procedure provides excellent quantitative reproducibility with experimental series standard deviations of <1% (mM), is nondestructive, and can be automated on demand.

## Introduction

Pretreatment and conversion of biomass into renewable energy and materials are a continuously expanding field of interest and research. Over the last decades, extensive research was performed, and literature studies were published addressing a great variety of conversion methods targeting biomass thermal liquefaction, pyrolysis, carbonization, and gasification.^1−4^ In general, the conversion methods and the resulting published papers have in common a focus on the target product stream for valorization, typically bio-oil or biochar, yet several pretreatment steps and conversion methods also result in an aqueous phase containing a significant proportion of biomass-derived products. These byproducts have been given only limited attention, and thus, there is a knowledge gap in the existing literature. Especially in a biorefinery context, identification and quantification of all byproducts are important to ensure sustainability and provide the basis for mass balance reports, monitoring product streams, and managing waste streams.

Various analytical procedures exist for identification and quantification of small organic molecules. These have been applied to samples from hydrothermal and thermochemical pretreatment of biomass, yet all analytical procedures possess weaknesses and limitations when targeting aqueous-phase samples. Chromatography is commonly used, typically reverse phase high-performance chromatography (HPLC). A great variety of HPLC procedures for aqueous-phase identification and quantification has been reported, using a refractive index detector (RID), an ultraviolet detector (UVD), and a diode array detector (DAD), coupled with mass spectrometry (MS) and two-dimensional comprehensive liquid chromatography coupled with DAD and MS (LC×LC/DAD-MS).^5−10^ Disadvantages of using analytical procedures involving HPLC involve their general dependency on previous information on sample content and extensive calibration curve preparations to identify and quantify sample compounds.

Gas chromatographic analyses coupled with mass spectrometry (GC–MS), which includes a method for separating and identifying ionized molecules in the MS detector, have limitations in that solvent delays exclude a great range of small molecules. Gas chromatography coupled with a flame ionization detector (GC-FID), in part, overcomes the solvent delay obstacle but precludes identification of components, and calibration curve preparations and signal overlaps due to similar chromatographic properties remain a general issue in chromatographic quantification methods.^11^ Hence, procedures using derivatization, i.e., substitution reactions on the sample compounds to change chemical structure and increase chromatographic detectivity, have been published and provide established procedures for overcoming loss of signals due to solvent delays and signal overlaps.^12−14^ Such methods require knowledge of compound functionalities to customize the derivatization agent. Due to potential for incomplete reaction, formation of multiple derivatives, and additional methodological complexity, which reduces the chromatographic reproducibility, derivatization is often a last resort in quantification procedures.^15^

The preparative laboratory and analytical protocol presented in this paper describes a comprehensive and precise method for rapid identification and quantification of organic molecules in aqueous product streams using quantitative nuclear magnetic resonance (qNMR) spectroscopy. The procedure has a wide applicability, and ^1^H NMR spectroscopy has the advantage of being directly quantitative and needing minimal sample preparation. ^1^H NMR is very well suited for quantification, as it gives a strong NMR signal due to the gyromagnetic ratio and high natural abundance, has reasonably short relaxation time, *T*_1_, and is present in most of the molecules of interest. However, since water itself has NMR-active protons, the water signal from the sample must be suppressed to avoid a signal overload. NMR procedures have been developed for this purpose in the context of metabolomics research and are now available in the standard libraries of the spectrometers.^16^

qNMR spectroscopy has been widely used for analysis of organic compounds at low concentrations in metabolomics, pharmaceuticals, and natural products multiple times over the years.^17−22^ In 2013, de Souza et al. reported on an NMR spectroscopy method for quantification and compositional analysis of polysaccharides.^23^ In 2009, Mittal et al. reported a method for quantitative analysis of sugars in wood hydrolysates.^24^ In 2017, Elliot et al. reported on NMR procedures on product samples from catalyzed conversion of xylose,^25^ and in 2019, Saito et al. published a review of the development of nuclear magnetic resonance as a tool of quantitative analysis for organic materials.^26^ In 2018, Yue et al. published a quantitative NMR study of process waters after furfural production from corncobs in China and process waters from subsequent hydrothermal carbonization of the same furfural production residues.^27^

The examples given in this paper address the composition of organics in the aqueous byproduct generated by steam explosion (STEX) of wood chips. The samples come from the production process of the patented energy-rich wood pellets Arbacore. Arbacore is produced by steam treatment of wood shavings in a reactor at elevated temperature followed by a subsequent rapid decompression of reactor pressure. This decompression of reactor pressure causes evaporation of water contained in the wood fibers and defibrillation/breakage of the wood fiber structure. The STEX technology used in the production process generates a moist solid material used for black Arbacore pellets and separates a considerable quantity of a condensed aqueous-phase effluent, rich in small organic compounds such as furfural and carboxylic acids.^28,29^ The concentration of the byproducts in the effluent depends on the severity of the thermal conditions, i.e., the temperature of the steam and the holding time before the pressure release.^30^

Different feedstocks give a unique composition profile due to the inherent simultaneous separation of treated biomass and condensation and collection of the aqueous process effluent after decompression of the reactor pressure. The need for analytical monitoring in this type of large-scale production is evident, and so, the main target of this study was to establish a protocol for the rapid workup, screening, and quantification of dissolved organic molecules using qNMR spectroscopy. This work also gives some representative quantitative results from Arbacore process effluents generated during a selected range of STEX processing conditions. The precision and repeatability of the analysis are evaluated, together with the reproducibility between instruments.

## Results and Discussion

Eight effluent samples from STEX runs are included in this study, and sample information is shown in Table 1. Effluent sample A was used as an analyte in the procedure and qNMR reproducibility tests for method verification while additionally including sample B during the instrument comparison. Samples I–VI were quantified in effluent quantification experiments.

**Table 1:** Experimental Large-Scale STEX Conditions^a^

sample	species loading (volume %)	filling time (s)	residence temp. (°C)	residence time (s)	residence pressure (bar)
A	50% Norway spruce (*Picea abies*) and 50% pine (*Pinus sylvestris*)	200	223 (±1)	500	21 (±1)
B		270			
I	100% Norway spruce (*Picea abies*)	270		0	20 (±1)
II				200	
III				400	
IV				600	
V				800	
VI				1000	

a The tabulated experimental conditions include time spent to fill roughly half of the preheated 11 m^3^ reactor before closing and exposing the wood shavings to residence temperature, time, and pressure inside the reactor. The process is terminated by explosive decompression.

Table 2 shows the results from control sample measurements, performed on furfural standards as method verification, where three sets of experimental parallels (six experiments) show deviations between prepared and measured concentrations of <0.6%. Table 3 documents the investigation of procedure reproducibility and presents the quantification of five repeated workups of sample A, displaying a standard deviation of <1.0% of each compound average, giving a σ < 1.9 mM. Table 4 shows the investigation of qNMR reproducibility and displays quantification of one workup of sample A analyzed repeatedly five times in different NMR tubes, resulting in a standard deviation of <0.4% of each compound average, giving a σ < 0.8 mM. For the comparison of different field strengths, seen in Table 5, the results give the quantification of two workups, each of samples A and B, analyzed at two field strengths, resulting in standard deviations of <1.0% of each compound average, giving a σ < 2.1 mM.

**Table 2:** Spectral and Quantification Data from Three Parallel Furfural Control Sample Sets (CS) Acquired at 600 MHz^a^

		concentration (μmol/g sample)					
	protons	CS.1.1	CS.1.2	CS.2.1	CS.2.2	CS.3.1	CS.3.2
dimethyl sulfone in the sample	6	43.2	43.2	43.1	43.0	43.1	43.1
furfural in the sample (prepared)		19.9	19.9	26.8	26.9	82.0	82.1
furfural in the sample (measured by qNMR)							
6.77 ppm	1	20.0	19.9	26.8	27.0	82.3	82.3
7.58 ppm	1	19.6	19.6	26.4	26.5	80.7	80.8
7.92 ppm	1	20.1	20.1	27.1	27.2	83.0	83.0
9.50 ppm	1	19.6	19.5	26.3	26.4	80.6	80.7
average furfural in the sample (measured by qNMR)		19.8	19.8	26.7	26.8	81.7	81.7
deviation (%)		0.4	0.5	0.2	0.5	0.4	0.6

a The internal standard integral was standardized to 6.000, and individual, post workup, dimethyl sulfone concentrations  were used in furfural concentration calculations.

**Table 3:** Spectral and Quantification Data from Five Workup Replicates of the Norway Spruce and Pine (1:1) Effluent, Sample A, Acquired at 600 MHz^a^

			integral					concentration (mM)					
compound identity	PPM	protons	A1	A2	A3	A4	A5	A1	A2	A3	A4	A5	σ (mM)
dimethyl sulfone	3.16	6	6.000	6.000	6.000	6.000	6.000	101.2	101.2	101.2	101.2	101.2	0.0
acetic acid	1.93	3	4.200	4.129	4.143	4.138	4.194	141.6	139.3	139.7	139.5	141.5	1.1
methanol	3.37	3	6.179	6.084	6.095	6.026	6.108	208.4	205.2	205.5	203.2	206.0	1.9
furfural	6.77	1	2.048	2.037	2.064	2.047	2.057	207.2	206.1	208.9	207.1	208.1	1.1
furfural	7.58	1	2.006	1.997	2.016	2.002	2.006	203.0	202.0	204.0	202.6	203.0	0.7
furfural	7.92	1	2.060	2.050	2.077	2.059	2.068	208.4	207.4	210.2	208.4	209.3	1.0
furfural	9.50	1	1.951	1.942	1.967	1.952	1.961	197.4	196.5	199.1	197.5	198.4	1.0
furfural average								204.0	203.0	205.5	203.9	204.7	0.9
formic acid	8.46	1	0.391	0.386	0.387	0.385	0.392	39.53	39.09	39.19	38.95	39.62	0.3

a The internal standard integral was standardized to 6.000.

**Table 4:** Spectral and Quantification Data from the Same Sample Workup of Norway Spruce and Pine (1:1), Sample A, Acquired in Five Sample Tubes at 600 MHz^a^

			integral					concentration (mM)					
compound identity	PPM	protons	A1.1	A1.2	A1.3	A1.4	A1.5	A1.1	A1.2	A1.3	A1.4	A1.5	σ (mM)
dimethyl sulfone	3.16	6	6.000	6.000	6.000	6.000	6.000	101.2	101.2	101.2	101.2	101.2	0.0
acetic acid	1.93	3	4.222	4.217	4.208	4.208	4.217	142.4	142.2	141.9	141.9	142.2	0.2
methanol	3.37	3	6.085	6.082	6.082	6.072	6.069	205.2	205.1	205.1	204.8	204.7	0.2
furfural	6.77	1	2.054	2.049	2.059	2.054	2.052	207.8	207.3	208.4	207.8	207.6	0.4
furfural	7.58	1	2.015	2.010	2.021	2.011	2.007	203.9	203.3	204.5	203.4	203.0	0.6
furfural	7.92	1	2.064	2.055	2.066	2.063	2.067	208.8	207.9	209.1	208.7	209.1	0.5
furfural	9.50	1	1.962	1.944	1.960	1.948	1.955	198.5	196.7	198.3	197.1	197.8	0.8
furfural average								204.8	203.8	205.1	204.3	204.4	0.5
formic acid	8.46	1	0.396	0.397	0.397	0.397	0.397	40.11	40.12	40.18	40.12	40.15	0.0

a The internal standard integral was standardized to 6.000.

**Table 5:** Spectral and Quantification Data from the Same Sample Tube of Norway Spruce and Pine (1:1) for Effluent Samples A and B, Acquired at 500 and 600 MHz^a^

			integral		concentration (mM)			integral		concentration (mM)		
compound identity	PPM	protons	A500	A600	A500	A600	σ (mM)	B500	B600	B500	B600	σ (mM)
dimethyl sulfone	3.16	6	6.000	6.000	101.2	101.2	0.0	6.000	6.000	101.2	101.2	0.0
acetic acid	1.93	3	4.347	4.327	146.6	145.9	0.5	3.439	3.433	116.0	115.8	0.2
methanol	3.37	3	6.191	6.281	208.8	211.8	2.1	4.668	4.701	157.4	158.5	0.8
furfural	6.77	1	2.144	2.152	217.0	217.8	0.6	1.697	1.705	171.7	172.5	0.5
furfural	7.58	1	2.111	2.105	213.6	213.0	0.4	1.681	1.668	170.0	168.8	0.9
furfural	7.92	1	2.163	2.178	218.9	220.4	1.1	1.716	1.721	173.6	174.1	0.4
furfural	9.50	1	2.099	2.100	212.4	212.5	0.1	1.674	1.673	169.4	169.2	0.1
furfural average					215.4	215.9	0.3			171.2	171.2	0.0
formic acid	8.46	1	0.412	0.412	41.7	41.7	0.0	0.319	0.319	32.2	32.3	0.1

a The internal standard integral was standardized to 6.000.

Both the furfural standards and the effluent analysis display excellent reproducibility using the described workup and analytical protocol. Low standard deviations and reproducible NMR spectra both verify the reliability of the analytical method, in addition to proving minimal system error. Chemical shifts were identical between all acquisitions, also confirming the method accuracy. The LOD (limit of detection) was determined to be around 0.3 μM (*S*/*N* = 2), while the LOQ (limit of quantification) is in the range of 3–30 μM, depending on which accuracy is required.

High field strength, such as the 850 MHz Bruker BioSpin Ascend NMR spectrometer used for preliminary identification procedures and inversion recovery pulse sequences as part of protocol determination, is not necessary for the quantification procedures. Table 5 shows that the 500 and 600 MHz field strengths display similar results, hence evidencing that a field strength of 500 MHz is adequate for these measurements. The 600 MHz NMR spectrometer with a QCI CryoProbe and four RF channels is both more expensive and would not be available for many users. Yet, the more conventional 500 MHz NMR spectrometer with a 5 mm BBO room-temperature probe is satisfactory for the reported procedure and is more widely available.

This reported laboratory protocol followed by NMR spectroscopy and subsequent quantification calculations is rapid and simple and can be automated in the case of large and frequent sample numbers. The procedure, including buffer addition and pH adjustments to prevent resonance influence from acidic protons and to ensure a reproducible chemical environment and chemical shifts (δ), provides an opportunity to consult spectral databases for identification purposes, and addition of the internal standard ensures the possibility for quantification. The method hence demands minimal sample information upon primary analysis and can thus be utilized for analysis of complex mixtures at an industrial scale. The protocol is a nondestructive quantification procedure, hence allowing sample and/or spectral re-analysis for identification and quantification of initially unidentified peaks at a later process or biorefinery (or other) developing stage.

**Figure 1:**
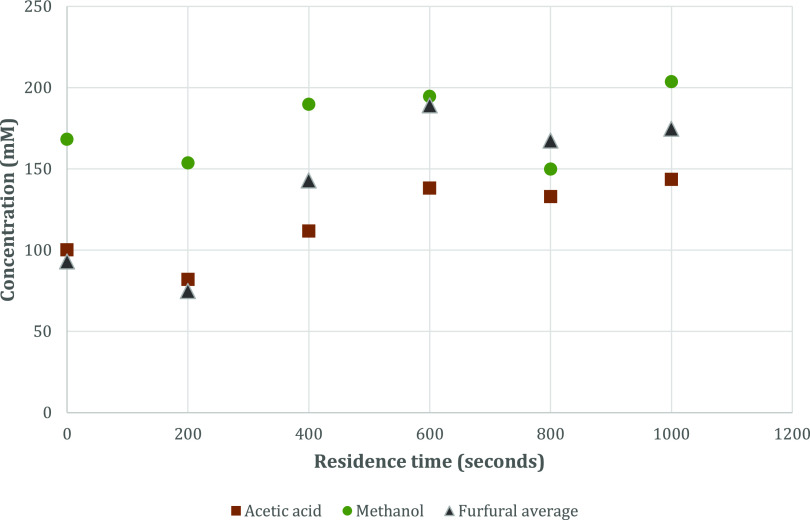
Concentrations of acetic acid, methanol, and furfural in effluent samples at increasing residence duration, samples I–VI from STEX of Norway pine. STEX conditions are given in Table 1. Numerical quantification data is given in Table S1 in the Supporting Information.

Figure 1 provides compositional data for processing effluents generated through a range of six STEX samples (I–VI) where the residence time before the pressure release is varied from 0 to 1000 s. Though this paper is not primarily aimed at giving a comprehensive quantitative study of processing effluents generated from large-scale STEX of wood shavings, the authors consider the elevated yields of formic acid, acetic acid, and furfural following the increased reactor residence time (200–600 s) particularly interesting followed by a leveling out or decrease upon further increase in residence time (600–1000 s). It is also noteworthy that the reactions producing furfural already have given significant product yields during the filling of the reactor, as seen from the data for a residence time of 0 s.

## Conclusions

This paper describes and reports on direct application of quantitative analytical NMR spectroscopy to investigate aqueous product streams, particularly targeting biorefinery byproducts. Preparative laboratory procedures combined with subsequent proton NMR spectroscopy with water signal suppression using presaturation pulses during relaxation delay, *noesygppr1d*, were established, evaluated, and verified in practice using three Bruker BioSpin NMR spectrometers; an 850 MHz AVANCE III HD with a 5 mm TCI CryoProbe, a 600 MHz AVANCE NEO with a QCI CryoProbe, and a conventional 500 MHz AVANCE with a 5 mm BBO room-temperature probe additionally confirmed the quantification method to be applicable. The reported preparative laboratory procedures combined with NMR spectroscopy for quantification purposes provide excellent reproducibility with experimental series standard deviations of <1% (mM), are nondestructive, and can be automated on demand. The utility of the procedure is demonstrated in a set of STEX effluent analyses, showing that the concentration of the major dissolved organic compounds increases with the residence time in the reactor from 0 to 600 s and then level out our decrease slightly.

## Experimental Section

### Materials and Reagents

All reagents and solvents were purchased from Merck KGaA (Darmstadt, Germany) and used without any further purification. All standard components are commercially available.

### Large-Scale Steam Explosion (STEX) Performed by Arbaflame AS

The effluent samples for analysis were provided by Arbaflame AS and collected from the Arbacore pellet-producing factory located at Grasmo (Akershus) in eastern Norway. A description of the technology is given in Wolbers et al. (2018).^31^ Samples from eight different STEX conditions with variable residence times were included in this study, and sample information is shown in Table 1. Effluent sample A was the analyte in the method verification tests given in Tables 3 and 4 while additionally including sample B in the instrument comparison tests in Table 5. The major dissolved organic species in samples I–VI were quantified, and the results are shown in the effluent quantification experiments in Figure 1.

### Preparation of NMR Samples

The internal standard (IS) used in this quantification procedure is dimethyl sulfone ((CH_3_)_2_SO_2_/DMSO_2_). Effluent samples are prepared for qNMR acquisition by using 8 mL of the condensed STEX effluent and adding 0.400 mL of a 2.125 M solution of dimethyl sulfone in distilled water (TraceCERT DMSO_2_). Target concentration of the internal standard in the sample at this stage is 0.1012 M, ensuring analysis within its optimal range of quantification.^32^ Spectral NMR signals of sample components and the IS should be of comparable height, which can be achieved by adjusting the concentration of the internal standard in the sample solution. A stock solution containing 0.010 M sodium phosphate dibasic dihydrate buffer (≥99.0% Na_2_HPO_4_·2H_2_O) and 20% deuterium oxide (99.9 atom % D D_2_O containing 0.05 wt % TSP (3-(trimethylsilyl)-propionic-2,2,3,3-d_4_ acid), sodium salt) was prepared and added to the sample ensuring a volume ratio of 1:1, hence giving the analyzed sample a 10% volume of deuterium oxide. pH was adjusted to 7.4 using a 1.0 M HCl or 1.0 M NaOH solution. Buffer addition and pH adjustments were made to prevent resonance influence from acidic protons and to ensure a reproducible chemical environment and chemical shifts (δ). All pH adjustments were performed using a Metrohm 798 MPT Titrino automatic titrator. The prepared sample (600 μL) was transferred to 5.0 mm × 7″ Wilmad 528 NMR tubes or 5.0 × 103.5 mm SampleJet NMR tubes depending on sampling acquisition. Volumetric accuracy was ensured by Eppendorf Research plus pipettes.

### NMR Spectral Acquisition

In quantitative NMR spectroscopy, it is crucial to ensure that all signals have relaxed fully between each transient. This means ensuring that all spins have reached equilibrium before applying a new pulse. Relaxation time, *T*_1_, was measured with an inversion recovery experiment. To ensure that all signals have reached equilibrium, a relaxation delay, *d*_1_, of at least five times the *T*_1_ of the slowest relaxing signal of interest is used, ensuring that 99.3% of the equilibrium magnetization (signal) is measured.^17,33^

Compounds of interest in the aqueous solution mixtures are quantified based on the internal standard method using the integral of dimethyl sulfone ((CH_3_)_2_SO_2_/DMSO_2_). The internal standard was included during the inversion recovery pulse sequence, at a field strength of 850 MHz.

Three different NMR spectrometers were used in this study, all from Bruker BioSpin, an 850 MHz AVANCE III HD equipped with a 5 mm TCI CryoProbe, a 600 MHz AVANCE NEO equipped with a QCI CryoProbe, and a 500 MHz AVANCE equipped with a 5 mm BBO room-temperature probe. For compound identification, effluent samples underwent workup according to the described protocol, and NMR samples were run using 850 MHz. For identification purposes, 1D ^1^H (*zgesgppe*), HSQC (*hsqcetgpsisp2.2*), and HMBC (*hmbcetgpl3nd*) spectra were acquired. Compound identification was aided by online databases (Biological Magnetic Resonance Data Bank and PubChem). The *T*_1_ relaxation of compounds of interest was measured using an inversion recovery experiment with solvent suppression using excitation sculpting with gradients.

For quantification purposes, ^1^H 1D NOESY with water suppression using presaturation, *noesygppr1d*, was used, as it is an acquisition technique of high-quality and reproducible spectra from aqueous samples.^34−36^ The spectra at 600 MHz were acquired at 298 K using a spectral width of 30 ppm, a time domain data size of 128k, 2 dummy scans, and 8 scans. The relaxation delay, *d*_1_, was set to 50 s, which was 5.5 times the longest measured *T*_1_ at 850 MHz (9 s). The spectra at 500 MHz were acquired using a spectral width of 30 ppm, a time domain data size of 64k, 4 dummy scans, and 8 scans.

### Accuracy and Reproducibility

Three sets of qNMR experiments were prepared and analyzed at 600 MHz as method verification to investigate and monitor protocol accuracy, as shown in Tables 2–4. One set of qNMR experiments was also run on both 500 and 600 MHz instruments to investigate field strength requirements, as given in Table 5.

### Quantification and Concentration Calculation

The most abundant compounds in the effluent samples were selected for quantification. They comprise formic acid (signal at 8.46 ppm), acetic acid (1.92 ppm), methanol (3.37 ppm), and furfural (6.77, 7.58, 7.93, and 9.51 ppm). The final furfural concentration is calculated as the average of its four signals. Integration regions for quantification were selected as the region around each signal, out to but not including ^13^C satellite signals.

All components were quantified based on internal standard dimethyl sulfone (DMSO_2_). NMR acquisition was engaged by Topspin 2.1 at 500 MHz, Topspin 4.0 at 600 MHz, Topspin 3.6 at 850 MHz, and IconNMR. NMR data were processed using a line broadening of 0.3 Hz, and signals were integrated (10–0 ppm) using TopSpin 4.0.7 software. Quantification of the sample components was performed by direct calculation from the resonance peak integrals, together with initial volumes of samples, IS concentration, molecular masses, certified purity of the reference standard (IS), and a normalization of the number of protons giving rise to the respective signals. Signals from labile protons, such as −OH and −NH_2_, are not considered in this quantification procedure.

The concentration of each component, *M*_A_, was calculated according to eq 1, where *I*_A_ is an integral of the component and *n*_A_ is the number of protons giving rise to the signal. *I*_DMSO_2__ is the integral of the DMSO_2_ signal, *n*_DMSO_2__ is the number of protons giving rise to the DMSO_2_ signal (6 protons), and *M*_DMSO_2__ is the concentration of DMSO_2_ in the NMR sample (101.2 × 10^–3^ M).^18^
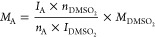
1

### Method Verification—Control Samples

Six furfural control samples (CS), furfural (≥98.5%) in distilled water (three parallel pairs), were prepared for qNMR acquisition according to the same protocol as effluent sample workup for method verification. Each parallel was worked up separately with individual addition of the internal standard and stock solution (buffer) and pH adjustment to 7.4. The qNMR spectra were acquired at 600 MHz in 5.0 × 103.5 mm SampleJet NMR tubes on the same day as protocol workup. Concentration calculations are based on mass (g) during workup and are shown in Table 2.

### Method Verification—Procedure Reproducibility

Condensed effluent sample A collected from large-scale STEX of Norway spruce and pine (1:1) was prepared five separate times according to the protocol, and qNMR spectra were acquired using 600 MHz in 5.0 × 103.5 mm SampleJet NMR tubes (experiments A1–A5). Sample preparation and acquisition were both performed on the same day for all five experiments. The quantification calculations were performed using eq 1 and are shown in Table 3.

### Method Verification—qNMR Reproducibility

An equivalent sample of condensed effluent sample A collected from large-scale STEX of Norway spruce and pine (1:1), as in the procedure reproducibility test, was prepared once according to the protocol, and qNMR spectra were acquired using 600 MHz from the same portion in five individual 5.0 × 103.5 mm SampleJet NMR tubes (experiments A1.1–A1.5). Sample preparation and acquisition of all five spectra were performed on the same day, and concentration calculations are shown in Table 4.

### Instrument Comparison

**Figure 2:**
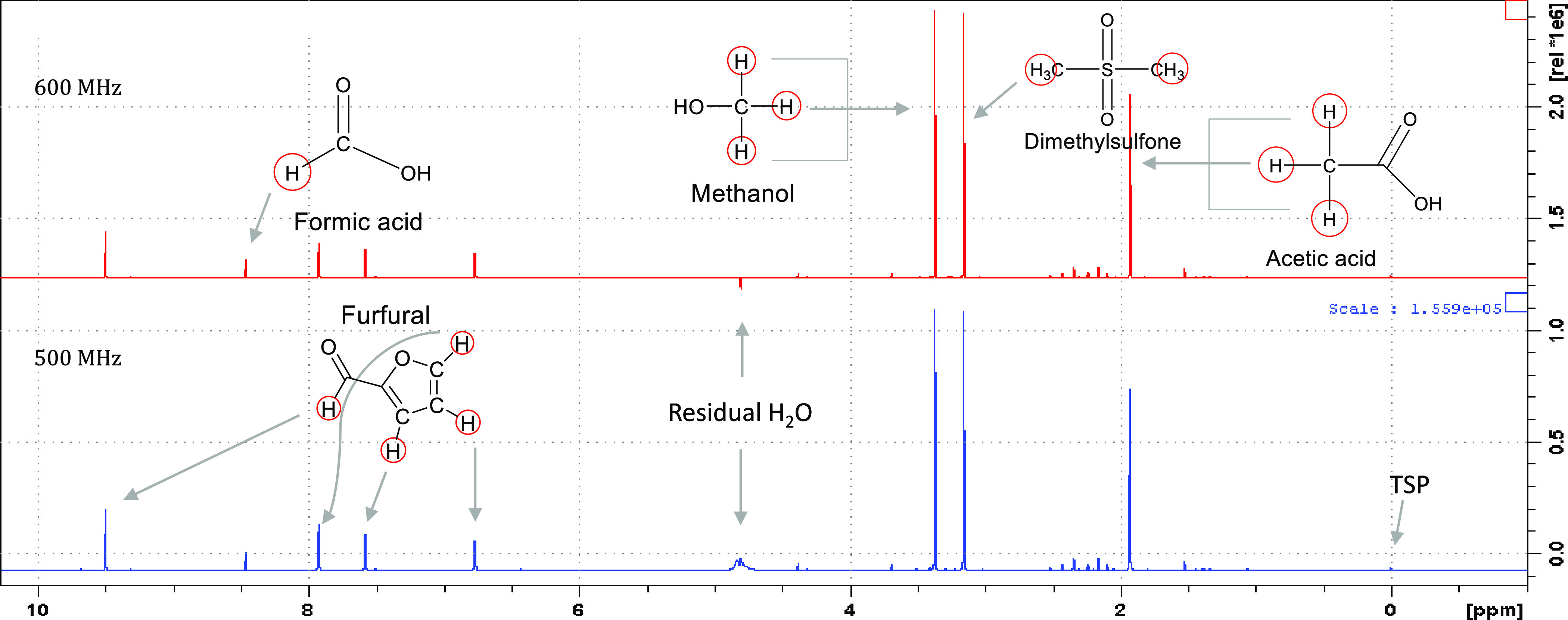
Stacked NMR spectra of effluent sample A at 500 and 600 MHz.

Two samples of effluents A and B, collected from large-scale STEX of Norway spruce and pine (1:1), were prepared according to the protocol, and qNMR spectra were acquired using both 500 and 600 MHz using the same respective 5.0 mm × 7″ Wilmad 528 NMR tube for each sample. Stacked NMR spectra for sample A are shown in Figure 2. The peak at 4.7 ppm is a residual water signal from the presaturated water suppression acquisition. Concentration calculations are shown in Table 5. Both samples were worked up, and qNMR spectra were acquired at 500 and 600 MHz on the same day from the respective sample tubes.

### Effluent Quantification Experiments

Six samples of the effluent, samples I–VI, collected from large-scale STEX of Norway spruce, were each prepared once according to the protocol, and qNMR spectra were acquired using 600 MHz in six individual 5.0 × 103.5 mm SampleJet NMR tubes. Sample workup and qNMR acquisition were both performed on the same day. The investigated parameter was residence time inside the reactor (0–1000 s, see Table 1), and the results are shown in Figure 1. Concentration calculations for quantification are shown in Table S1 in the Supporting Information.

## Supporting Information Available

(Table S1) numerical data for effluent quantification experiments of samples I–VI (PDF)

## References

[ref1] Pang S. Advances in thermochemical conversion of woody biomass to energy, fuels and chemicals. Biotechnol. Adv. 2019, 37, 589–597. 10.1016/j.biotechadv.2018.11.004. 30447327

[ref2] Nanda S.; Mohammad J.; Reddy S. N.; Kozinski J. A.; Dalai A. K. Pathways of lignocellulosic biomass conversion to renewable fuels. Biomass Convers. Biorefin. 2014, 4, 157–191. 10.1007/s13399-013-0097-z.

[ref3] Bridgwater A. V. Renewable fuels and chemicals by thermal processing of biomass. Chem. Eng. J. 2003, 91, 87–102. 10.1016/S1385-8947(02)00142-0.

[ref4] Wertz J.-L.; Bédué O. Lignocellulosic biorefineries; EPFL Press: Lausanne, Switzerland, 2013; 10.1201/b15443.

[ref5] Becker R.; Dorgerloh U.; Paulke E.; Mumme J.; Nehls I. Hydrothermal Carbonization of Biomass: Major Organic Components of the Aqueous Phase. Chem. Eng. Technol. 2014, 37, 511–518. 10.1002/ceat.201300401.

[ref6] Lazzari E.; Arena K.; Caramão E. B.; Herrero M. Quantitative analysis of aqueous phases of bio-oils resulting from pyrolysis of different biomasses by two-dimensional comprehensive liquid chromatography. J. Chromatogr. A 2019, 1602, 359–367. 10.1016/j.chroma.2019.06.016. 31227362

[ref7] Lazzari E.; dos Santos Polidoro A.; Onorevoli B.; Schena T.; Silva A. N.; Scapin E.; Jacques R. A.; Caramão E. B. Production of rice husk bio-oil and comprehensive characterization (qualitative and quantitative) by HPLC/PDA and GC × GC/qMS. Renewable Energy 2019, 135, 554–565. 10.1016/j.renene.2018.12.053.

[ref8] Chen H.; Qin L.; Yu B. Furfural production from steam explosion liquor of rice straw by solid acid catalysts (HZSM-5). Biomass Bioenergy 2015, 73, 77–83. 10.1016/j.biombioe.2014.12.013.

[ref9] Dubuis A.; Le Masle A.; Chahen L.; Destandau E.; Charon N. Centrifugal partition chromatography as a fractionation tool for the analysis of lignocellulosic biomass products by liquid chromatography coupled to mass spectrometry. J. Chromatogr. A 2019, 1597, 159–166. 10.1016/j.chroma.2019.03.031. 30922725

[ref10] Tomasini D.; Cacciola F.; Rigano F.; Sciarrone D.; Donato P.; Beccaria M.; Caramão E. B.; Dugo P.; Mondello L. Complementary Analytical Liquid Chromatography Methods for the Characterization of Aqueous Phase from Pyrolysis of Lignocellulosic Biomasses. Anal. Chem. 2014, 86, 11255–11262. 10.1021/ac5038957. 25327521

[ref11] Panisko E.; Wietsma T.; Lemmon T.; Albrecht K.; Howe D. Characterization of the aqueous fractions from hydrotreatment and hydrothermal liquefaction of lignocellulosic feedstocks. Biomass Bioenergy 2015, 74, 162–171. 10.1016/j.biombioe.2015.01.011.

[ref12] Madsen R. B.; Jensen M. M.; Mørup A. J.; Houlberg K.; Christensen P. S.; Klemmer M.; Becker J.; Iversen B. B.; Glasius M. Using design of experiments to optimize derivatization with methyl chloroformate for quantitative analysis of the aqueous phase from hydrothermal liquefaction of biomass. Anal. Bioanal. Chem. 2016, 408, 2171–2183. 10.1007/s00216-016-9321-6. 26804738

[ref13] Madsen R. B.; Bernberg R. Z. K.; Biller P.; Becker J.; Iversen B. B.; Glasius M. Hydrothermal co-liquefaction of biomasses–quantitative analysis of bio-crude and aqueous phase composition. Sustainable Energy Fuels 2017, 1, 789–805. 10.1039/C7SE00104E.

[ref14] Kim K. R.; Hahn M. K.; Zlatkis A.; Horning E. C.; Middleditch B. S. Simultaneous gas chromatography of volatile and non-volatile carboxylic acids as *tert*.-Butyldimethylsilyl derivatives. J. Chromatogr. A 1989, 468, 289–301. 10.1016/S0021-9673(00)96323-4.

[ref15] Choi C. K.; Dong M. W. 5 - Sample Preparation for HPLC Analysis of Drug Products. In Separation Science and Technology; Ahuja S., Dong M. W., Eds.; Elsevier: United Kingdom, 2005; Vol. 6, pp. 123–144.

[ref16] Giraudeau P.; Silvestre V.; Akoka S. Optimizing water suppression for quantitative NMR-based metabolomics: a tutorial review. Metabolomics 2015, 11, 1041–1055. 10.1007/s11306-015-0794-7.

[ref17] Holzgrabe U. Quantitative NMR spectroscopy in pharmaceutical applications. Prog. Nucl. Magn. Reson. Spectrosc. 2010, 57, 229–240. 10.1016/j.pnmrs.2010.05.001. 20633364

[ref18] Bharti S. K.; Roy R. Quantitative 1H NMR spectroscopy. TrAC, Trends Anal. Chem. 2012, 35, 5–26. 10.1016/j.trac.2012.02.007.

[ref19] Yamazaki T.; Eyama S.; Takatsu A. Concentration Measurement of Amino Acid in Aqueous Solution by Quantitative ^1^H NMR Spectroscopy with Internal Standard Method. Anal. Sci. 2017, 33, 369–373. 10.2116/analsci.33.369. 28302980

[ref20] Rundlöf T.; McEwen I.; Johansson M.; Arvidsson T. Use and qualification of primary and secondary standards employed in quantitative ^1^H NMR spectroscopy of pharmaceuticals. J. Pharm. Biomed. Anal. 2014, 93, 111–117. 10.1016/j.jpba.2013.09.010. 24206940

[ref21] Liang X.; Du L.; Su F.; Parekh H. S.; Su W. The application of quantitative NMR for the facile, rapid and reliable determination of clindamycin phosphate in a conventional tablet formulation. Magn. Reson. Chem. 2014, 52, 178–182. 10.1002/mrc.4048. 24464591

[ref22] Burton I. W.; Quilliam M. A.; Walter J. A. Quantitative ^1^H NMR with External Standards: Use in Preparation of Calibration Solutions for Algal Toxins and Other Natural Products. Anal. Chem. 2005, 77, 3123–3131. 10.1021/ac048385h. 15889900

[ref23] de Souza A. C.; Rietkerk T.; Selin C. G. M.; Lankhorst P. P. A robust and universal NMR method for the compositional analysis of polysaccharides. Carbohydr. Polym. 2013, 95, 657–663. 10.1016/j.carbpol.2013.02.036. 23648027

[ref24] Mittal A.; Scott G. M.; Amidon T. E.; Kiemle D. J.; Stipanovic A. J. Quantitative analysis of sugars in wood hydrolyzates with 1H NMR during the autohydrolysis of hardwoods. Bioresour. Technol. 2009, 100, 6398–6406. 10.1016/j.biortech.2009.06.107. 19674893

[ref25] Elliot S. G.; Tolborg S.; Sádaba I.; Taarning E.; Meier S. Quantitative NMR Approach to Optimize the Formation of Chemical Building Blocks from Abundant Carbohydrates. ChemSusChem 2017, 10, 2990–2996. 10.1002/cssc.201700587. 28627762

[ref26] Saito T.; Yamazaki T.; Numata M. Development of nuclear magnetic resonance as a tool of quantitative analysis for organic materials. Metrologia 2019, 56, 054002 10.1088/1681-7575/ab348d.

[ref27] Yue F.; Pedersen C. M.; Yan X.; Liu Y.; Xiang D.; Ning C.; Wang Y.; Qiao Y. NMR studies of stock process water and reaction pathways in hydrothermal carbonization of furfural residue. Green Energy Environ. 2018, 3, 163–171. 10.1016/j.gee.2017.08.006.

[ref28] Brusletto R.; Kleinert M. Method of producing carbon-enriched biomass material. US10119088B2, 2018.

[ref29] Arbaflame AS The future of renewable energy is here. 2016, Arbaflame AS, viewed 19 July 2019, http://www.arbaflame.no/arbacore/.

[ref30] Overend R. P.; Chornet E. Fractionation of lignocellulosics by steam-aqueous pretreatments. Philos. Trans. R. Soc., A 1987, 321, 523–536. 10.1098/rsta.1987.0029.

[ref31] Wolbers P.; Cremers M.; Robinson T.; Madrali S.; Tourigny G. Biomass pre-treatment for bioenergy – Case study 4: The steam explosion process technology; IEA Bioenergy: 2018.

[ref32] Sigma-Aldrich Co. QUANTITATIVE NMR - Technical Details and TraceCERT Certified Reference Materials, Sigma-Aldrich Co.. 2017, 3050 Spruce St., St. Louis, MO 63103.

[ref33] Oxford University Quantitative NMR Spectroscopy. 2017, Oxford University, viewed 2 July 2019, http://nmrweb.chem.ox.ac.uk/Data/Sites/70/userfiles/pdfs/quantitative-nmr.pdf.

[ref34] Abreu A. C.; Fernández I. NMR Metabolomics Applied on the Discrimination of Variables Influencing Tomato (*Solanum lycopersicum*). Molecules 2020, 25, 3738 10.3390/molecules25163738. PMC7463728 32824282

[ref35] Allwood J. W.; De Vos R. C. H.; Moing A.; Deborde C.; Erban A.; Kopka J.; Goodacre R.; Hall R. D. Chapter sixteen - Plant Metabolomics and Its Potential for Systems Biology Research: Background Concepts, Technology, and Methodology. In Methods in Enzymology; Jameson D., Verma M., Westerhoff H. V., Eds.; Academic Press: 2011; Vol. 500, pp. 299–336. 10.1016/B978-0-12-385118-5.00016-5 21943904

[ref36] Ross A.; Schlotterbeck G.; Dieterle F.; Senn H. Chapter 3 - NMR Spectroscopy Techniques for Application to Metabonomics. In The Handbook of Metabonomics and Metabolomics; Lindon J. C., Nicholson J. K., Holmes E., Eds.; Elsevier Science B.V.: Amsterdam, 2007; pp. 55–112.

