# Supplementary material for: A Workup Protocol Combined with Direct Application of Quantitative Nuclear Magnetic Resonance Spectroscopy of Aqueous Samples from Large-Scale Steam Explosion of Biomass
Source: ACS Omega. 2021 Mar 2;6(10):6714–21. doi: 10.1021/acsomega.0c05642 (PMC7970479; doi:10.1021/acsomega.0c05642)
Supplement: Supplementary file 1 — ao0c05642_si_001.pdf [file ao0c05642_si_001.pdf]

Supporting Information

A workup protocol combined with direct application of quantitative NMR-spectroscopy of aqueous samples from large-scale steam explosion of biomass

Camilla Løhre*, Jarl Underhaug, Rune Brusletto and Tanja Barth

Department of Chemistry, University of Bergen, Allégt. 41, 5007 Bergen, Norway:

*correspondence camilla.lohre@uib.no (C.L);

jarl.underhaug@uib.no (J.U.);

tanja.barth@uib.no (T.B.)

Arbaflame AS, Henrik Ibsens gate 90, 0255 Oslo, Norway:

rune@arbaflame.no (R.B)

Table S1 – Effluent quantification experiments of samples I-VI. STEX parameters enabling effluent production were kept at 270 seconds reactor filling time, residence temperature of 223 (±1) °C, residence pressure of 20 (±1) bar and varying reactor residence time of 0 (I), 200 (II), 400 (III), 600 (IV), 800 (V) and 1000 (VI) seconds.

**Compound identity**			**Integral**						**Concentration (mM)**					
	**PPM**	**Protons**	**I**	**II**	**III**	**IV**	**V**	**VI**	**I**	**II**	**III**	**IV**	**V**	**VI**
Dimethyl sulfone	*3.16*	*6*	*6.000*	*6.000*	*6.000*	*6.000*	*6.000*	*6.000*	*101.2*	*101.2*	*101.2*	*101.2*	*101.2*	*101.2*
Acetic acid	1.93	3	2.969	2.428	3.310	4.091	3.942	4.256	100.1	81.9	111.6	138.0	132.9	143.5
Methanol	3.37	3	4.985	4.555	5.626	5.768	4.439	6.036	168.1	153.6	189.7	194.5	149.7	203.6
Furfural	6.77	1	0.858	0.692	1.355	1.803	1.587	1.633	86.8	70.0	137.1	182.4	160.6	165.2
Furfural	7.58	1	0.906	0.728	1.409	1.841	1.650	1.718	91.6	73.6	142.5	186.3	166.9	173.8
Furfural	7.92	1	0.946	0.760	1.448	1.918	1.694	1.774	95.7	76.9	146.5	194.0	171.4	179.5
Furfural	9.50	1	0.970	0.781	1.439	1.908	1.686	1.778	98.2	79.0	145.6	193.1	170.6	179.9
Furfural average	-	-	-	-	-	-	-	-	93.1	74.9	142.9	189.0	167.4	174.6
Formic acid	8.46	1	0.354	0.287	0.375	0.440	0.415	0.436	35.9	29.0	38.0	44.5	42.0	44.1
